# Numerical and Experimental Performance Analysis of the Chirped Fiber Bragg Grating Based Abrasion Sensor for the Maintenance Applications in the Industry 4.0

**DOI:** 10.3390/s20030770

**Published:** 2020-01-31

**Authors:** Konrad Markowski, Kacper Wojakowski, Ernest Pokropek, Michał Marzęcki

**Affiliations:** Institute of Telecommunications, Warsaw University of Technology, Nowowiejska 15/19, 00-665 Warsaw, Poland; kacp.woja@gmail.com (K.W.); er.pokropek@gmail.com (E.P.); M.Marzecki@tele.pw.edu.pl (M.M.)

**Keywords:** fiber Bragg grating, fiber optic sensor, abrasion sensor, maintenance, Industry 4.0

## Abstract

In this article, an extensive analysis of the performance of the fiber optics-based abrasion sensor that utilizes chirped fiber Bragg grating, is presented. For the response investigation during abrasion, a numerical analysis, based on the transfer matrix method and coupled mode theory, is provided. The influence of the SLED source spectral position in respect to the spectral position of the chirped fiber Bragg grating is evaluated together with the influence of the changes of the ambient temperature of the sensor. Experimental verification of the sensor’s performance is provided, together with the proposition of the packaging of the sensor. In the article, a simple, cost-effective and multiplexation-ready concept of the wear or abrasion sensor system is presented and discussed.

## 1. Introduction

Wear monitoring of tools, devices and industrial installation is a crucial problem in progressing automation combined with the fourth industrial revolution [1,2,3], where device maintenance is one of the most important assumptions. Mass production often expects diagnostic tools for wear monitoring that enable the operation of the device or factory production line without any work interruption. In the Industry 4.0 revolution prognosis, it is stated that devices will be able to self-diagnose and determine the moment for component replacement for the proper maintenance of the given device [4]. Information collected during the process of the device utilization will then be used to improve production processes, which will enable optimization of the production and resources utilization more efficiently [5]. It is predicted that automation revolution initiated in factories will cover more and more new areas, moving from factories to houses and cities by the technology of smart homes and cities. All these elements are connected by the idea of Internet of Things (IoT). According to this idea, in the future all everyday objects will feel due to sensors and communicate freely with each other through wireless networks [6,7,8,9]. In the context of these changes, it becomes necessary to develop increasingly new wear sensors that can be used in various applications and can be an integral part of devices or buildings. Currently, wear sensors are being used in installations that are exposed to damage under the influence of corrosive and toxic compounds, in mining machines, and production lines where downtime at work generates high costs [10,11,12,13]. Such sensors are used for monitoring and diagnostics in order to obtain optimal working conditions at which the wear of the elements is relatively low and the work efficiency is the highest possible. The large amount of data collected by the sensors allows for a better understanding of the factors affecting the operation of equipment and entire production systems. Equipment manufacturers can use this information to further improve the construction of those machines. The final goal of this progress is to develop machines able to self-improve. A great amount of emphasis in current research has been placed on the optimization of production processes in terms of material consumption as well as their greater flexibility for modifications for other parameters. Therefore, in the future, machines will be constantly modified and adapted to current conditions using special actuators. These modifications will occur under the influence of collected data from many sensors, including wear and abrasion sensors. 

Information on tool wear can be obtained indirectly from information on temperature, pressure, speed, time of use, pressure or vibration. The synthesis of data from several sensors [14] in some cases allows for a fairly accurate determination of the degree of tool’s wear, however, this requires extensive prior research and the determination of multi-parametric functions for a specific tool without a guarantee of compatibility for tools with a modified shape, material or alloy. A variety of methods have already been developed to record wear or damage of tools. One of the methods is based on recording of the acoustic wave and vibrations generated by the tool [15]. Using this technique, it is possible to analyze many different events that occurs during the work of the device. The frequency and amplitude of the vibrations depends in this case on the nature of their source. The received signal is a combination of vibrations from various elements of a tool or machine, such as a bearing or blade. The analysis of this signal allows for precise determination of a vibration source based on the frequency, amplitude and nature of vibrations [16]. Another popular technique is thin-film technique based on resistive abrasion lines. In this type of sensor, resistive lines exposed to wearing factors increase their surface resistivity. Such sensors can be made through the utilization of classic techniques such as photolithography or by additive printing techniques, like for example screen printing [17,18]. Another type of wear sensor is material thickness sensors recording the wear of a tool or abrasive element. This type of sensor can be based on electro-magnetic fields [19], radiographic methods [20,21], system of lenses [22] or optical fiber sensors [23,24,25,26,27,28]. However, for specific applications, utilization of the classical electronic sensor is impossible. This is due to the fact, that in applications, where big electrical engines are utilized, typically, one may expect high electromagnetic fields. Thus, for such applications, it is more convenient to utilize a fiber-based wear monitoring system. While through that the problem of electromagnetic interference is eliminated, typically, sensors that are based on the optical systems are far more expensive, when compared to the classical electronics. Thus, to provide an alternative fiber-based solution, development of the cost effective optical sensor for abrasion or wear monitoring is required.

In this study, the fiber optics wear sensor with chirped fiber Bragg grating (CFBG) has been investigated. The main idea behind this study was to provide a cost effective, easy-to-implement system that is based on power monitoring. For optimization, a theoretical analysis of the operation of the sensor has been provided through utilization of the numerical model of the CFBG that is excited with a super-luminescent diode (SLED). Experimental analysis of the operation of the sensor has also been provided, together with a discussion over the achieved results. 

## 2. Sensing Principle of Sensor

**Figure 1:**
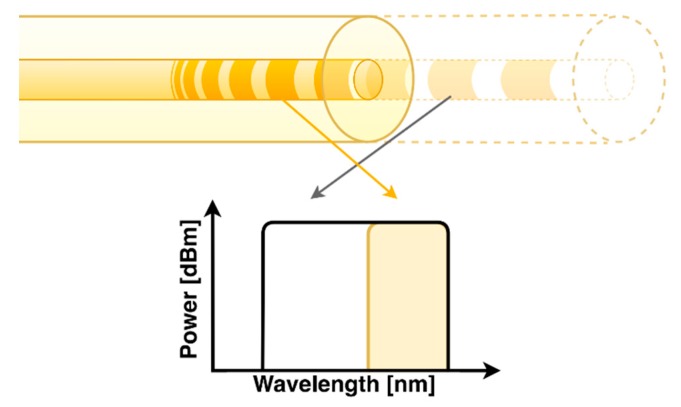
Scheme, showing the relation between the spatial position of the grating and reflected signal.

The principle of the operation of the presented sensor has been described in [26]. When the length of the chirped grating decreases, due to the corrosion of the surface in which the sensor has been perpendicularly placed, its reflection decreases as well. In turn, when the length of the grating drops continuously, spectral reflection width drops as well. As a result, if the grating is placed at the end of the fiber, a reduction of this length will also result in a reduction of the total reflected power from the grating. Figure 1 shows schematically the relation between the spatial position of the grating and corresponding Bragg wavelength.

While the method itself allows for precise measurements, it also requires utilization of the expensive measurement equipment such as of Optical Spectrum Analyzer (OSA) or any other measurement unit that allows for spectral analysis. Moreover, the method described in [26] also require utilization of the processing unit that will compare the normalized signal to the numerically calculated reflection spectra. However, since grating reflects wideband SLED, the power received by the measurement equipment also decreases. Thus, it is proposed that measurement can be performed not only by means of the reflection width of the optical signal, but also by means of the power at the receiver.

Let us assume that SLED generates a gaussian-like wideband signal, which can be described through its full width at half maximum (FWHM). For such a case, normalized power versus wavelength of the SLED can be written as:

(1) $$P_{\mathrm{SLED}}\left(\lambda \right)=P_{0}\frac{\sqrt{ln2}}{w\sqrt{\pi }}e^{\frac{-4{\left(\lambda -\lambda_{0}\right)}^{2}}{w^{2}}}$$

where *w* states for a FWHM, λ_0_ is the central wavelength of the of the signal that is being generated by SLED, while P_0_ is the peak power density of the source.

The most common and easiest method for modeling Bragg grating with non-uniform responses is based on the Transfer Matrix Method (TMM), together with Coupled Mode Theory (CMT) [29]. For such an approach, it is assumed that the field amplitude a_M_ is entering a structure, which can be described using a matrix notation. Since Equation (1) describes the power density of the generated signal, the amplitude of the electric field can be written as its square root. Thus, the amplitude of light that is generated by SLED a_M_,_SLED_(λ) will take the following form

(2) $$a_{M,\mathrm{SLED}}\left(\lambda \right)=\sqrt{P_{\mathrm{SLED}}\left(\lambda \right)}$$

With such a definition of the field amplitude that enters the grating, the boundary conditions for TMM also must be adjusted, resulting in a reflection of the grating that can be described as a R_SLED_(λ), which is a function related to the spectral width of the SLED source. As a result, normalized, to total power of SLED, signal that is being reflected from the grating can be written in terms of the following equation:

(3) $$P_{\mathrm{NORM}}=\frac{\int_{-\infty }^{+\infty }R_{\mathrm{SLED}}\left(\lambda \right)d\lambda }{\int_{-\infty }^{+\infty }P_{\mathrm{SLED}}\left(\lambda \right)d\lambda }$$

**Figure 2:**
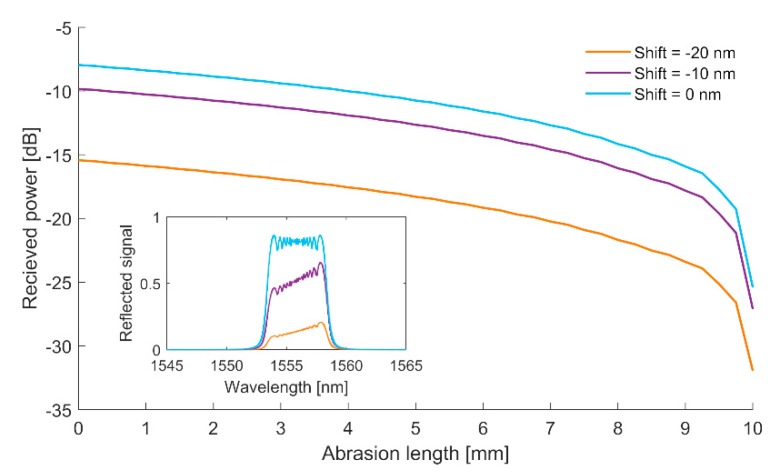
Received power versus the length of the grating for several mutual positions of the center of the Bragg grating reflection and SLED generation. Initial Bragg grating length, of which the spectral response is presented in the inset graph, is assumed to be 10 mm.

Let us now assume that λ_0,CFBG_ is a Bragg wavelength that corresponds to the center of the chirped Bragg grating reflection. This central wavelength can then be associated with a center wavelength of SLED generation. Such an aspect is of crucial importance, due to the fact that this spectral region is related to the highest power density of the SLED source. Thus, when received power is used as a parameter that determines the length of the grating, the mutual position of these two wavelengths clearly will affect the measurement. Numerical analyses that show the value of the received power versus the length of the grating, for several mutual positions of both the central wavelength of the grating and central wavelength of the SLED, are presented in Figure 2. It is assumed that P_0_ is equal to 0 dBm, while the source has 25 nm FWHM, with the central wavelength equal to 1550 nm. The corresponding Bragg grating length is assumed to be 10 mm, while a phase mask chirp that has been employed for manufacturing of this grating is constant and equal to 0.35 nm/mm.

As it can be observed, when the grating becomes shorter, the total reflected power from the grating drops. However, the change in mutual position of the center of the grating reflection and wavelength of SLED does not affect the slope of the curve. Thus, the factor that determines the resolution of the measurement is related to the receiver sensitivity not the spectral position of the grating. However, the presented method does not allow precise measurement of the abrasion length and can be employed in the system where a certain threshold of the abrasion can be indicated.

**Figure 3:**
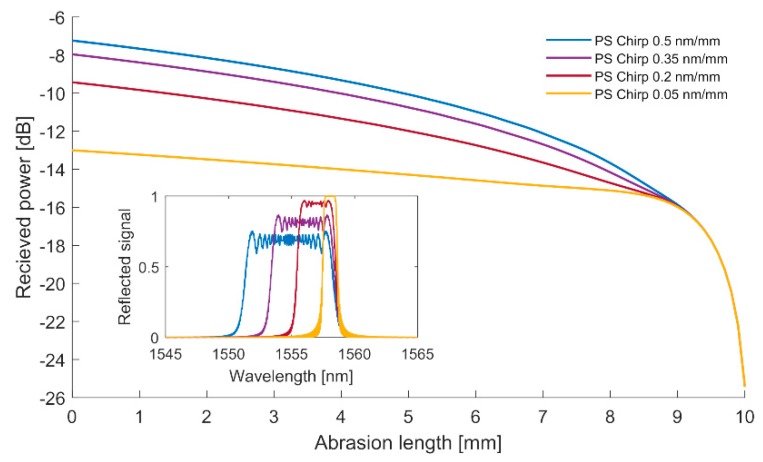
Received power versus the length of the grating for several values of fringe pattern chirp of the gratings. The central wavelength of the SLED corresponds to the center of the Bragg grating reflection. Initial Bragg grating length, of which the spectral response is presented in the inset graph, is assumed to be 10 mm.

The next factor that has to be taken into account is a value of the phase mask chirp. Let us assume the same spectral properties of the source as for the previous numerical analysis. Moreover, let us assume that the mutual position of the center wavelength of the grating and center wavelength of the SLED source is equal to each other, while the grating length is constant and equal to 10 mm. Figure 3 shows a change in the received power versus grating length for several values of the phase mask chirp.

Together with the higher phase mask chirp, the dynamics of the abrasion sensor increase. For the case where chirp is equal to 0.5 nm/mm, the drop in the received power in the range 0–8 mm is equal to 8 dB, while for 0.05 nm/nm this value equals only 1 dB. Thus, for certain applications (for instance, when only an indication of a certain threshold is required), it is more convenient to use gratings with low chirp, or even uniform ones. However, precise measurement of the material damage requires gratings that have been manufactured with a phase mask of an as high as possible chirp. 

Last but not least, due to the fact that the Bragg wavelength varies with temperature, the relationship between ambient temperature and the performance of the sensor has to also be taken into account. As reported in numerous studies [30], Bragg wavelength shift of the grating depends on both thermo-optic effect and thermal expansion. In turn, it can be stated that temperature sensitivity of the standard grating written in SMF-28 fiber is equal to 11.3 pm/°C. This aspect can be taken into account through a change proportional to the ΔT change of the detuning component in CMT equations [29,30].

**Figure 4:**
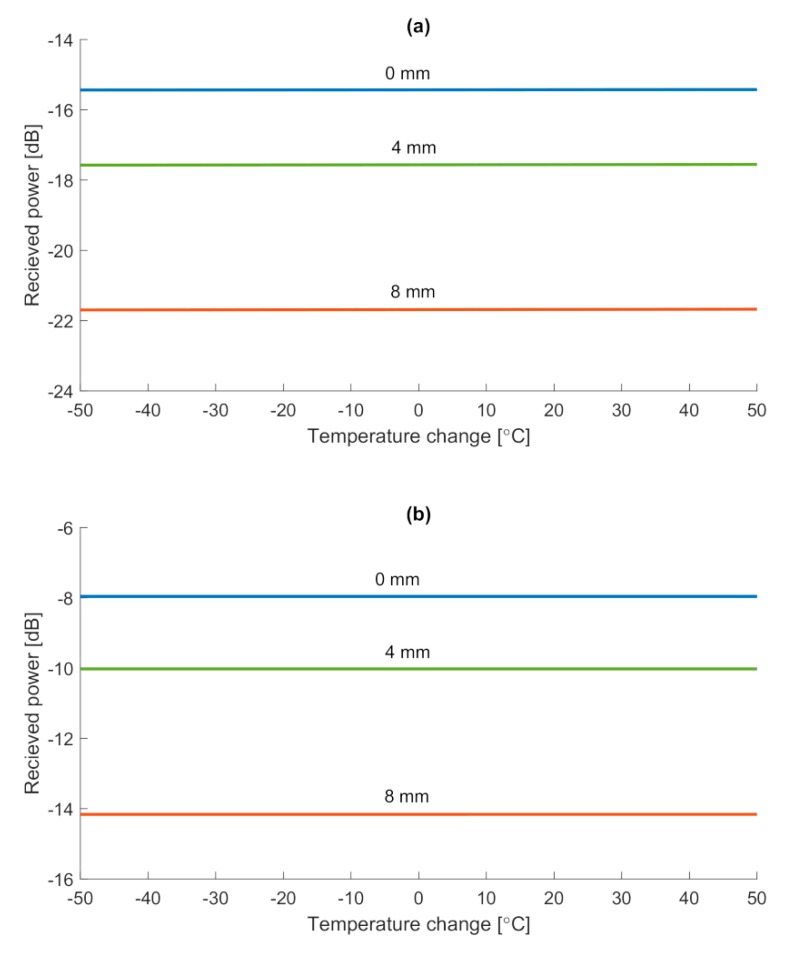
Received power versus the ambient temperature change of the grating for several values of grating length. The central wavelength of the SLED (**a**) is mutually shifted by 20 nm and (**b**) corresponds to the central wavelength of the grating. Initial Bragg grating length is assumed to be 10 mm.

Thus, let us assume two cases, first, in which central Bragg wavelength corresponds to the center wavelength of the SLED source, and the second, where the mutual position of the center wavelength of the grating and SLED source is shifted by a factor of 20 nm, while the center wavelength of the grating is lower than the center wavelength of the source. The chirp of the fringe pattern of the grating for the presented case has been set to 0.35 nm/mm. Numerical results are presented in Figure 4.

What can be stated, is a fact, that while the grating shifts its position due to the changes in ambient temperature, received power remains almost constant. Simulation shows that for ±50 °C temperature change, received power changes at most by a value of 0.1 dB. In turn, it can be concluded that grating as an abrasion sensor is temperature insensitive. The presented numerical analysis proves that such a sensor can be utilized for damage monitoring together with low-cost equipment, i.e., a standard photodetector. What is more, on the basis of the results from Figure 2, it can be stated that a sensor network, working on a single SLED source, can be designed, without the need for a complex calibration process for each sensor. Such a sensor network then could be utilized for monitoring of the degradation process of the structure at multiple points.

**Figure 5:**
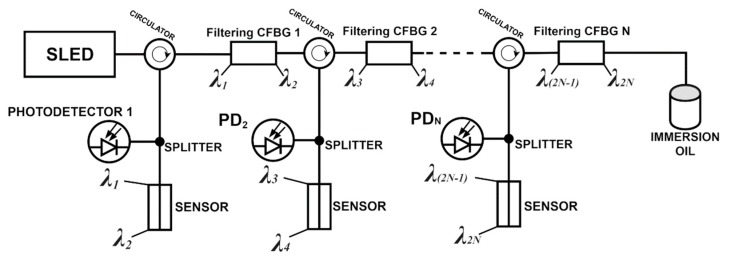
Scheme for abrasion sensor network that is based on CFBG—λ_(2N-1)_ indicates initial wavelength that is being reflected from the CFBG N grating structure, while λ_N_ is last wavelength that grating reflects.

Nevertheless, for achieving as high dynamics of the measurement as possible, it is crucial to eliminate unwanted back reflections of the whole signal. This requires utilization of the immersion oil, together with filtering Bragg grating in which the reflection width is matched with the reflection width of the utilized sensor. The concept of such a sensing network is presented in Figure 5. What has to be underlined is the fact that the utilization of the photodetector in such a sensor network can be as high as several kHz, without the need for utilization of expensive spectrophotometers such as OSA. 

## 3. Experimental Analysis

### 3.1. Experimental Setup

**Figure 6:**
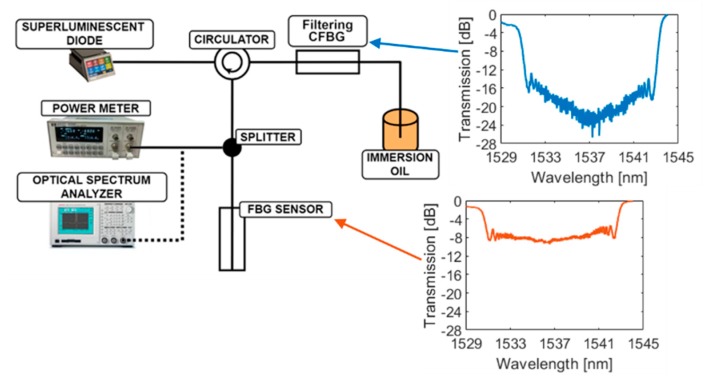
Experimental measurement setup of the abrasion sensor. Connections are made with SMF-28 optic fiber. Inset graphs show transmission spectra for both sensor and filtering grating.

For experimental purposes, two CFBGs have been written in hydrogen-loaded SMF-28 fiber and hydrogen-loaded GF3 photosensitive fiber, respectively, using a Coherent BraggStar Industrial-LN excimer laser, through a 25-mm-long chirped phase mask with a central period of 1061 nm and a chirp ratio equal to 0.35 nm/mm. The total length of the sensor grating was 24 mm, while for filtering grating, the grating length was 25 mm, resulting in a slightly higher reflection width. Figure 6 shows an experimental setup for the measurement of the reflected signal parameters during simulated abrasion of the proposed sensor. As a filtering CFBG a grating written in SMF-28 has been utilized, while for the sensor CFBG a stronger grating written in GF3 photosensitive fiber has been used. This allowed for achieving high spectral power density of the signal that has been reflected from the sensor grating, allowing for measurements of the total reflected power from the sensor CFBG with higher dynamics. The average reflectivity over the spectral band of the sensor grating was as high as 90%, while for the filtering grating it was around 70%.

The spectral transmission, measured through utilization of the flat supercontinuum source of the filtering and sensor grating, is indicated in Figure 6. As it can be noted, the spectral width of the filtering grating is slightly higher than the one that corresponds to the sensor grating. This enables utilization of the abrasion sensor in the conditions, where the ambient temperature of these gratings is different in respect to the temperature sensitivity of the CFBG, which is approximately equal to 11.3 pm/°C, as it has been denoted previously. Since for our case the temperature conditions were stable, this difference is as low as possible, allowing for measurements where the ambient temperature difference of these gratings may be as low as ±20 °C. 

**Figure 7:**
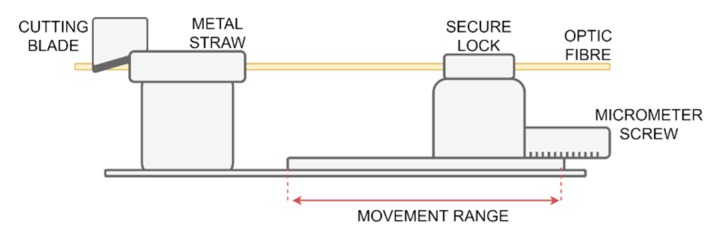
Experimental setup of the wearing simulation diagram.

First, the signal from the InPhenix SLED IPSDD1504 with FWHM of 65 nm and 5dBm mounted on a ThorLabs CLD1015 was transferred to the circulator, and then sent to the filtering fiber Bragg grating, in order to cut out the narrow signal band that is matched with the spectral width of the abraded grating. To minimize unwanted back Fresnel reflection, the end of the fiber with the filtering grating was submerged in the immersion oil, in which the refractive index was equal to N_o_=1.515 at room temperature, as is noted in Figure 6. The reflected photonic signal from CFBG then went back into the circulator and was transferred to the splitter, where one part of the signal goes on the FBG sensor and the reflected wave travels back to either the power meter (PM) or optical spectrum analyzer (OSA). As a PM, Hewlett Packard 8153A Lightwave Multimeter PM (sensitivity from +27 to −90 dBm) was used along with the Advantest Q8384 OSA (wavelength range 600 to 1700 nm). To simulate the abrasion process, the setup denoted on Figure 7 has been employed. The sensor was cut mechanically, i.e., using a cutting blade, which was mounted on XYZ precise micrometer screws. This allowed for proper alignment of the abrasion sensor with the axis of the cutting blade. The fiber itself was attached to the single axis translation stage and was moved towards the cutting blade using another micrometer screw. The art of the FBG meant to be cut was sticking out of the small metal straw that had an inner diameter equal to 200 ± 20 μm attached to another immovable stand, placed within a short distance from the first one. For each measurement, the FBG was moved and cut for 1 mm ±0.3 mm, resulting in a total of 26 measurements of 25 mm FBG. 

### 3.2. Measurements 

**Figure 8:**
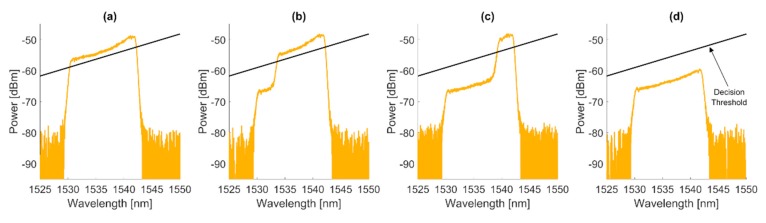
Sensor’s spectrum for various lengths. The first (**a**) and last (**d**) figure are the spectrum without any length reduction and after full abrasion, respectively. The second (**b**) and third (**c**) figure show the spectrum after the fourth (4 mm) and seventeenth (17 mm) cut, respectively. The decision threshold (black line) is the reference line from which the spectrum width was measured.

**Figure 9:**
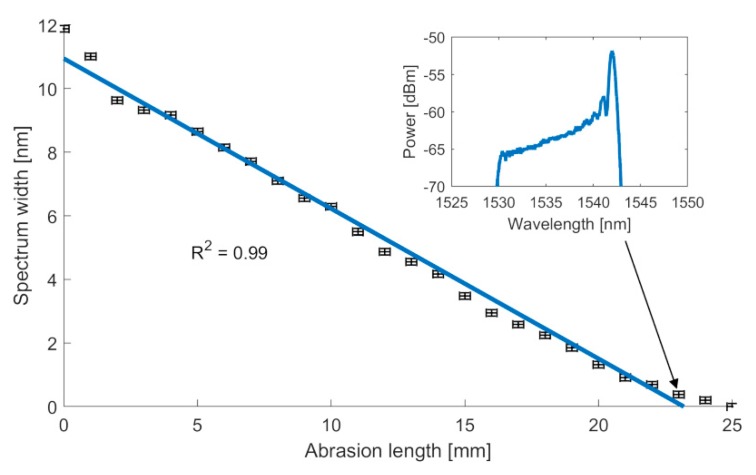
Spectrum width—abrasion relation graph.

From the OSA, clear, visible data has been obtained, showing the reduction of the FBG spectral width, which is a direct indication of the abrasion that occurred. What is important is that clearly the spectrum that is related to the filtering grating becomes visible at a noise level of −65 dBm ±5dBm, depending on the wavelength, while the rest of the SLED spectrum is as low as −80 dBm. The level of −80 dBm is similar to the natural noise level of the OSA for the chosen equipment settings, thus it may be stated that all unwanted Fresnel back reflections from the end of the fiber are eliminated. Several spectra measurements are shown consequently in Figure 8a–d, indicating both the spectral response of the FBG sensor for several lengths and the decision threshold for spectral width measurements that are aligned with the spectral response of the SLED. The resulting reduction of the spectral width of the FBG sensor is presented in Figure 9.

For the chirped fiber Bragg gratings, the grating period is linearly dependent on the reflected spectrum length. Reducing the grating length by cutting off its parts, consequently results in a smaller reflected spectrum width. The spectral width of the grating is calculated on the basis of the decision threshold. The slope of this threshold can be calculated as a tangent for the point that corresponds to the center wavelength of the non-abraded grating. A constant term for this threshold can then be calculated for the measurement point that corresponds to the wavelength of the maximum reflectivity reduced by 4 dB. This in turn prompts linear dependence of the spectral width of the grating and its length. However, it has to be noted that for the proposed method of measurement, for lengths of the grating that are smaller than 4 mm, this linearity is corrupted. Such a simple method for measuring the spectral width of the grating, that can be easily employed in the microprocessor unit, does not take into account the issue of the physical nature of the chirped Bragg grating written in the fiber. Namely, when the total length of the CFBG is lower than several values related to the chirp of the grating, the nature of the quasi-uniformity is revealed [31,32]. In turn, for this region, the relation between spectral width and the length of the grating ceases to be monotonic, and the precision of the sensor drops. The spectral response for the case where grating becomes quasi-uniform is presented in the inset of Figure 9.

For the presented structure, the spectrum width as a linear function of abrasion can be estimated as

*s(x) = −0. 4720x + 10.9439* (4)

Where s is the spectrum width in nm, while x is the abrasion length in mm.

In this function, the constant term is equal to the reflected spectrum width, and the slope is close to −0.5, as the FBG is chirped linearly, resulting in an almost inversely proportional relation. The s-intercept, however, is not exactly equal to the actual reflected spectrum width, as the best fit slope does not cross the first data point.

The RMSE value for the line approximation is equal to 0.4 nm, while mean absolute error (MAE) is equal 0.3 nm; thus, the key factor of error are the first and last measurements, deviating slightly from the line characteristics, due to previously discussed quasi-uniformity of the short CFBGs.

On the basis of the resolution of the OSA, which in our case was ±0.01 nm, the resolution of the abrasion sensor has been estimated to be as low as ±0.02 mm. The value of the resolution strictly depends on the chirp of the grating and becomes lower for the gratings that have been manufactured through utilization of the phase mask with a higher chirp.

For the measurement of the total reflected power from the grating, a crucial limitation in terms of the noise floor is related to the spectrum that is reflected from the filtering grating. Along with the decreased length of the sensor FBG, such a noise floor becomes visible, as it has been denoted in Figure 8d. The reason for such a state is due to the value of the Fresnel back reflection from the sensor FBG that cannot be neglected, because a reduction of the grating’s length is in its nature non-deterministic. As a result, it has to be taken into account that the surface end of the sensor FBG may be smooth, which is similar to the surface of the fiber that has been cut with the fiber cleaver. Resulting from such a fact, the uncertainty of the measurement has been estimated to be equal to ±0.3 dB. Due to the typical high-power stability of the SLED sources, the error related to the SLED power variation has been neglected. 

**Figure 10:**
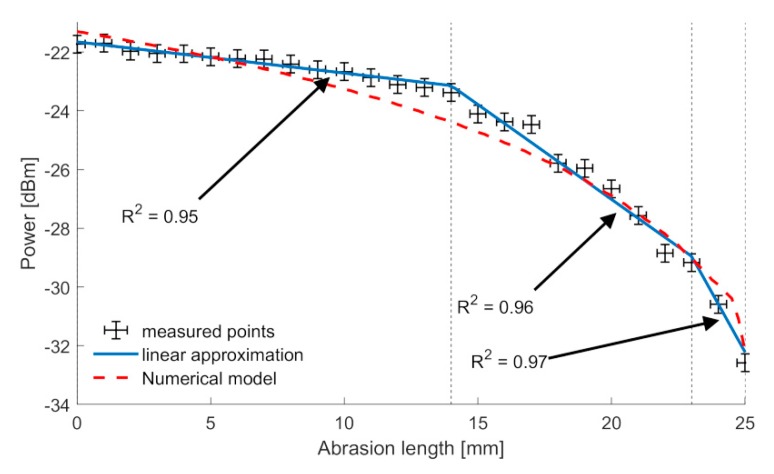
Abrasion–reflectance relation graph.

The relation between received power and an abrasion length is presented in Figure 10.

As it has been presented, the numerical model described is coincident with the experimental measurements. However, for the real application, due to the existence of the noise, by means of Fresnel reflection it is necessary to calibrate the sensor response in terms of approximation functions. Obtaining the power to abrasion relation in Figure 10, the approximate function can be deduced in two ways, either by using an approximation of a polynomial of third degree or using a piecewise linear approximation. For the obtained experimental data, the polynomial approximation is as follows:

*R(x) = − 0.0012x^3^ + 0.0196x^2^ − 0.1827x − 21.6268* (5)

For given intervals, the linear function approximations are as follows:

*[0,14] : R1(x) = −0.1062x − 21.6566* (6)

*[14,23] : R2(x) = −0.6459x − 14.1003* (7)

*[23,25] : R3(x) = −1.6338x + 8.6210* (8)

Where, x stands for abrasion length in mm, while p is received power in dBm. 

Which, in turn, means that the sensitivity-derivative of power on consecutive intervals is equal to −0.1062, −0.6459, and −1.6338.

The root mean square error (RMSE) value for the polynomial approximation is equal to 0.2 dB, while for the linear ones it is 0.3 dB (across all intervals), which is consistent with the estimated value of the error that results from unwanted Fresnel back reflections from the end of the sensor FBG. Therefore, both of these methods can be considered reliable to depict the relation between power and abrasion as functions. 

Through calculating derivatives from the obtained functions, and thus their rate of change, the sensitivity of the sensor depending on the abrasion level is obtained. As the magnitude of abrasion increases or the length of the FBG drops, the derivative of function of abrasion becomes steep, and thus the sensor is more sensitive to any further changes. This experimental analysis is matched with the numerical results presented in Section 2.

For the presented Figure 6 setup, the total resolution of the measurement can be calculated as ±0.4 mm for the range indicated in (5), ±0.07 mm for the range indicated in (6), and ±0.03 mm for the range indicated in (7). The resulting resolution strictly depends on the resolution of the PM utilized in the experiment, which for our case was estimated to be equal to ±0.04 dB. 

## 4. Results and Discussion

**Figure 11:**
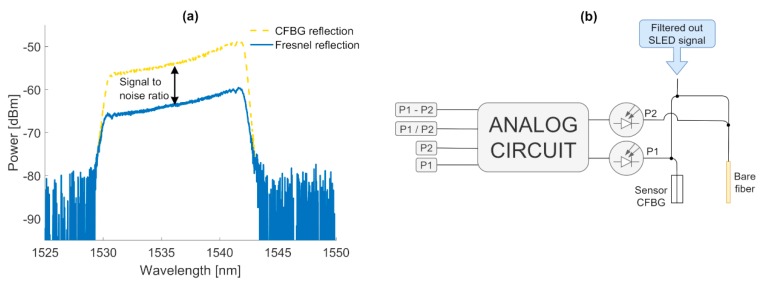
**(a**) Reflected signal from the grating, together with an indication of the Fresnel reflection and (**b**) the system setup for monitoring of the Fresnel reflection.

As it has been presented in the previous section of the manuscript, together with the drop of the sensor length, the amount of received power drops as well. However, an important aspect that is related to the wideband reflection from the end of the grating has to be taken into account. While the dynamics of the proposed approach are improved through utilization of the filtering CFBG, the residual noise that is related to the wideband Fresnel reflection from the end of the fiber occurs either way, which can be observed in Figure 8d. Typically, abrasion or wear sensors are utilized in the environments where the surface damage occurs due to the friction between two surfaces and is not deterministic in its nature. As a result, this will disrupt the measurement indication of the damaged surface. The ratio between Fresnel reflection and the photonic signal can be treated as a signal-to-noise ratio, which is indicated in Figure 11a. To improve the robustness of the proposed approach, it is suggested to measure Fresnel reflection through utilization of the reference fiber that is placed right next to the sensor CFBG. The proposed architecture of the measurement system is presented in Figure 11b. 

When the filtered-out light enters the first 3dB coupler, it is divided proportionally between the fiber either with the CBFG sensor or bare one. As it has been presented previously, the amount of power that is being reflected from the sensor CFBG will drop together with its length. However, from the end of the bare fiber, the total reflected power will be irrespective of the abrasion length and will vary with accordance to the Fresnel reflection. Then, the two photodetectors will either measure the reflected signal from the sensor CFGB or from the end of the bare fiber. These two values of power could then be properly processed, providing information about the difference and ratio between these signals, as well as the independent information about these power levels.

Let us denote P_2_ and P_1_ as a Fresnel reflection from the bare fiber and reflection from the sensor CFBG, respectively. For P_1_, the total reflected power will be the sum of the reflection from the sensor CFBG and wideband Fresnel reflection, while for P_2_, it will depend only on the wideband Fresnel reflected signal. For the given definition, P_1_, P_2_, difference between P_1_ and P_2_ and ratio between P_1_ and P_2_ can be written as:

*P_1_ = 0.5P_FLITERED_(Ref_CFBG_(z) +F)* (9)

*P_2_ = 0.5P_FLITERED_F* (10)

*P_1_ − P_2_ = 0.5P_FLITERED_ (Ref_CFBG_(z) + F) − 0.5P_FLITERED_ F = 0.5P_FLITERED_ Ref_CFBG_(z)* (11)

*P_1_/P_2_ = 0.5P_FLITERED_ (Ref_CFBG_(z) + F)/ 0.5P_FLITERED_ F = Ref_CFBG_(z)/F +1* (12)

where, Ref_CFBG_(z) stands for the power that is reflected from the CFBG versus its length, and F stands for the Fresnel reflection, while P_FILTERED_ stands for the signal from the SLED that is filtered out through filtering CFBG.

Clearly, from Equation (11) it can be concluded that the measurement is irrespective of Fresnel reflection. However, these two fibers needs to be placed next to each other and it needs to be assumed that the Fresnel reflection from the end of these fibers is similar. On the other hand, by taking into account Equation (12), one can eliminate any power fluctuations of the SLED source, allowing for utilization in the measurement system of a low-cost uncooled wideband source of high-output optical power together with a low-cost photodetector. 

**Figure 12:**
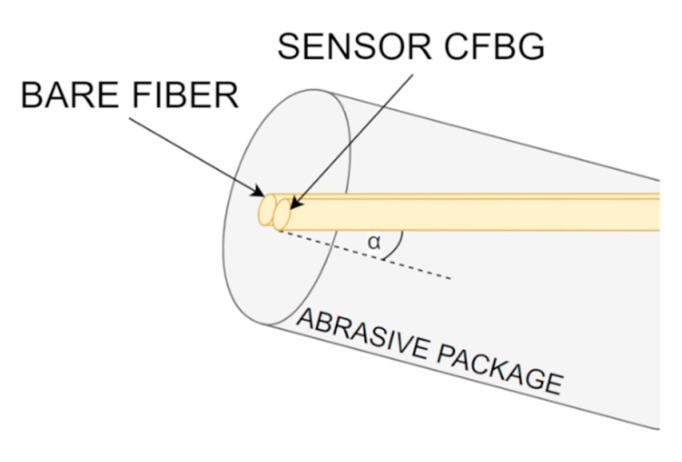
Abrasion sensor packaging together with Fresnel reflection reference, allowing for minimizing the unwanted Fresnel reflection from the end of the sensor grating.

While the presented approach allows for elimination of all unwanted variables in the measurement system, one needs to take into account the possibility of the formation of Fabry-Perot resonance. Such resonance can occur if the reflection from the tip of the sensor CFBG will be sufficiently high, resulting in distortion of the measured power. Thus, it is crucial to keep the Fresnel reflection on the possible lowest level. For that, in Figure 12, a proposition for the packaging of such a sensor is presented. Instead of placing the fibers in perpendicular position to the surface that is being wear down, it is suggested to place them at a certain angle, denoted as α. Thus, even if on the surface fiber tips perfectly reflective, for instance, metal layer will form, most wideband Fresnel reflection will be scattered, providing robust operation for the proposed scheme. However, one needs to take into account the reduction of the sensor measurement range and its resolution. 

To further improve the operation of the sensor, in the future it is suggested to utilize multicore fiber [33], together with the selectively written gratings. As a result, this will not only allow for achieving a greater operation range of the sensor (through inscription of the CFBG of the same spectral response but with different positions in respect to each other), but it will also allow to integrate a Fresnel reflection reference arm in the single fiber optic. As a result, the described sensor and its operation would become more compact and reliable.

Another important aspect when it comes to the utilization of CFGB as an abrasion sensor refers to its parameters stability over a time period. Before utilization of the sensor in the real system, grating, after manufacturing, needs to be annealed, through a heating process to stabilize its spectral parameters [34]. Thus, under the assumption that the grating will operate below 100 °C, it is necessary to anneal the grating in a temperature of at least 150 °C for 48 h.

## 5. Conclusions

In this paper, a simple and cost-effective method for damage monitoring that is based on a CFBG abrasion sensor has been presented. Both numerical and experimental analyses have been conducted, where the possible read out from the sensor was carried either through an OSA or a PM.

It has been shown that the measurement of the surface abrasion can be carried out either by utilization of the spectrophotometer (in this case, an OSA) or by utilization of the power meter. Numerical analysis of the impact of the mutual position of the center wavelength of the CFBG versus the center wavelength of the SLED source has been carried out, proving that sensitivity, by means of the change in the received power, is shift-independent. Moreover, a detailed analysis of the chirp ratio influence on sensitivity has also been carried out. The numerical analyses also proved that the presented method that is based on the measuring of the total reflected power from the grating is also irrespective of the small variation of the sensor ambient temperature.

The experimental part has been carried out for both concepts where total reflected power and spectral width have been measured. The quasi-uniform nature of the sensor has been discussed, while for the concept where the power meter has been utilized, it has been proven that in the proposed scheme, the highest sensitivity of the sensor can be achieved for the highest values of abrasion. 

Depending on the application, the sensor can be used in various ways. Taking its length into consideration, for thicker materials, a longer sensor (FBG) can be produced, and multiple of them can work simultaneously or independently. However, for real life application, the main issue that will limit the sensor performance will be related to the unwanted Fresnel reflection from its back. Thus, for future prospects, proper packaging of the sensor needs to be developed. Moreover, it is suggested that the emplacement of the sensor versus the surface subjected to the abrasion should not be perpendicular, and the influence of the emplacement angle on the value of the unwanted Fresnel back reflection should also be considered together with utilization of the reference bare fiber for direct Fresnel reflection monitoring. For further development of such a sensor, it is suggested to consider a multicore fiber, together with selective writing of fiber Bragg gratings technology.
